# Clutter Suppression Method for Off-Grid Effects Mitigation in Airborne Passive Radars with Contaminated Reference Signals

**DOI:** 10.3390/s21196339

**Published:** 2021-09-23

**Authors:** Yaqi Deng, Wenguo Li, Saiwen Zhang, Fulong Wang, Weichu Xiao, Zhi Cui

**Affiliations:** 1 College of Information and Electronic Engineering, Hunan City University, No. 518, Yingbin East Road, Yiyang 413000, China; dengyaqi@hncu.edu.cn (Y.D.); zhangsaiwen@hncu.edu.cn (S.Z.); caoyanqing@hncu.edu.cn (F.W.); zhicui@hnu.edu.cn (Z.C.); 2 College of Electrical and Information Engineering, Hunan University, No. 2, Lushan South Road, Yuelu District, Changsha 410082, China; weichuxiao@hnu.edu.cn

**Keywords:** passive radar, airborne radar, clutter suppression, off-grid effect, reference signal

## Abstract

For an airborne passive radar with contaminated reference signals, the clutter caused by multipath (MP) signals involved in the reference channel (MP clutter) corrupts the covariance estimation in space-time adaptive processing (STAP). In order to overcome the severe STAP performance degradation caused by impure reference signals and off-grid effects, a novel MP clutter suppression method based on local search is proposed for airborne passive radar. In the proposed method, the global dictionary is constructed based on the sparse measurement model of MP clutter, and the global atoms that are most relevant to the residual are selected. Then, the local dictionary is designed iteratively, and local searches are performed to match real MP clutter points. Finally, the off-grid effects are mitigated, and the MP clutter is suppressed from all matched atoms. A range of simulations is conducted in order to demonstrate the effectiveness of the proposed method.

## 1. Introduction

Passive radars, which utilize existing commercial sources as emitters of opportunity, offer advantages of low cost and strong survivability than compared to active radars [1,2]. Airborne passive radars apply passive radar technology on an airborne platform, providing the additional benefits of reduced terrain masking effect and improved detection abilities [3,4,5,6]. However, the motion of the platform causing ground clutter has angle and Doppler frequencies in airborne passive radars, which makes it challenging for conventional one-dimensional methods to separate targets from clutter.

Space-time adaptive processing (STAP) is a key tool for clutter suppression in airborne passive radar [7,8], where the reference signal is exploited for covariance matrix estimation. However, the traditional STAP requires a large number of independent and identically distributed training snapshots. It is difficult to collect the sufficient samples in heterogeneous environments. In addition, the high complexity in the computation of the high-dimensional covariance matrix inversion restricts the applicability of STAP. Many suboptimal STAP algorithms have been proposed to address these issues. Reduced-dimension STAP [9,10,11] and reduced-rank STAP [12,13] can reduce the number of required snapshots to twice of the reduced dimension or twice of the clutter rank. The training data selectors [14] can improve the target detection ability in heterogeneous environments with dense outliers. Recently, knowledge-aided STAP has demonstrated enhanced detection performances with minor training support by exploiting the prior knowledge [15,16,17]. Lately, Sparse representation technology has been widely considered in various fields [18,19], which encourages research on sparse-aware STAP. Sparse-aware STAP reconstructs the clutter covariance matrix by using sparse representation techniques, improving suppression capability and offering high-resolution imagery in a deficient-training-sample situation [20,21,22]. Assuming that the reference signal is pure, the aforementioned STAP algorithms can provide the desirable suppression performance in the airborne passive radar. However, such condition is hard to meet in the real environments. A contaminated reference signal results in a clutter snapshot consisting of two parts: one is the matched result of scatterers echoing with the direct-path (DP) signal, i.e., DP clutter; and the other is with multipath (MP) signals contaminated in the reference channel, i.e., MP clutter. As a result, targets located at the MP clutter area are suppressed and cancels DP clutter and MP clutter simultaneously when using the aforementioned STAP algorithms.

The cascaded method (CM), which performs the MP clutter suppression before STAP cancels DP clutter, can provide an alternative method for target detection in airborne passive radars with contaminated reference signals. Several MP clutter suppression methods have been developed [23,24,25]. In the modified blind equalization method, by exploiting the prior knowledge of the Doppler frequency of the DP signal, MP signals are restrained, and the reference channel is equalized [23]. However, the precise prior knowledge is often difficult to obtain in practice, and errors in prior information disturb the equalization quality for this method. Sparse reconstruction algorithms (SRA) for MP clutter suppression have been proposed in [24,25], where a range-Doppler dictionary is designed, a cost function with a sparse constraint is derived, and SRAs, such as *L*_1_-based recursive least squares, *L*_1_-based least mean square, and *L*_1_-based exponentially forgetting window least mean square (*L*_1_-EFWLMS), are used to solve the optimization problem. However, a gap between MP clutter patches and the predefined range-Doppler grids, which is also called the off-grid problem, is hardly avoided in these methods. Under such circumstances, the estimation performance of MP clutter degrades significantly. To the best of our knowledge, very little studies have been performed to overcome the off-grid problem in MP clutter suppression for airborne passive radar. The off-grid issue also arises in other applications such as sparse-aware STAP [26,27] and direction-of-arrival [28,29,30]. However, most methods are either ad hoc [31] or computationally intensive or both, and they cannot be directly applied in MP clutter suppression for airborne passive radar.

This paper focus on the off-grid problem in MP clutter suppression for airborne passive radar. In the proposed suppression method, the off-grid effects are mitigated by performing a global search and several local searches in each iteration. The global search is similar to the estimation method in SRA, while selected atoms are closer to the real location of MP clutter than SRA because of the additional local search steps. The procedures of CM based on the proposed algorithm are summarized as follows. First, the global range-Doppler dictionary is designed under the framework of the existing SRA from which the global atoms that are most relevant to the residuals are selected. Second, an iterative local search is performed around the selected atoms to match the real MP clutter points. Then, the above operation is repeated until the predetermined criterion is satisfied, and MP clutter is suppressed from all matched atoms. Finally, the DP clutter is suppressed by the traditional STAP. Compared with the existing SRA, the proposed algorithm exhibits better detection performance in the presence of off-grid problem.

The remainder of this paper is organized as follows. In Section 2, based on the geometry of airborne passive radar systems, the signal model and problem statement are presented. In Section 3, the proposed MP clutter suppression method for off-grid effects mitigation is detailed, and CMs based on the proposed algorithm are summarized. Subsequently, the effectiveness of the proposed approaches is verified by using a range of simulations in Section 4. Finally, relevant conclusions are presented in Section 5.

Notation: In this paper, the symbols ⊙ and ⊗ denote the Hadamard product and the Kronecker product, respectively. The operations of transposition and conjugate transposition are denoted by ${\left(\cdot \right)}^{Τ}$ and ${\left(\cdot \right)}^{H}$, respectively. $1_{A\times B}$ is the A×B matrix of which all elements are 1, the operator Re{⋅} selects the real part of the argument, and E{⋅} denotes the expected value operation. Additionally, ${\left\|\cdot \right\|}_{1}$ and ${\left\|\cdot \right\|}_{2}$ denote the *l*_1_-norm and *l*_2_-norm operations, respectively.

## 2. Signal Model

**Figure 1:**
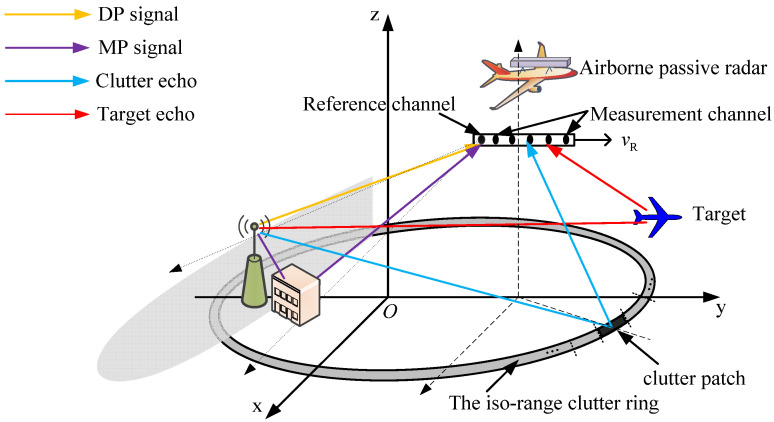
Bistatic geometry of airborne passive radar.

Without loss of generality, the system under consideration is the airborne passive radar where a ground-based non-cooperative source is utilized as the transmitter of opportunity. The bistatic geometry of the airborne passive radar system is shown in Figure 1. The reference signal is received by the reference antenna, which is directed toward the direction of the transmitter, and a uniform linear array consisting of *N* elements receives the measurement signal. The reference signal is contaminated by *N_T_* MP signals, and the time bins of DP and MP signals are *l*_d_ and *l_p_*, *p* = 1, 2, …, *N_T_*, respectively. During the coherent processing interval, the received reference signal and measurement signals are segmented into *M* equivalent pulses, with a pulse repetition interval of *T*_r_. After matched filtering for the radar returns from *M* pulses and *N* channels by using reference signal, the received data are stored as an L×M×N data-cube where *L* is the total number of range cells. By stacking a slice of the data cube, which corresponds to the received data at one range cell, an MN×1 vector termed as a space-time snapshot is formed.

Neglecting the effect of range ambiguities, the clutter patches contributing to the clutter snapshot at a given range cell are located only on one iso-range clutter ring [7]. Considering a contaminated reference signal, the clutter snapshot consists of two parts [24]. The first part is DP clutter. The DP clutter snapshot $x_{dc,l}$ at range cell *l* is obtained when the scatterer echoes with time bin (*l + l_d_*) are passed through the matched filtering with the DP signal. Therefore, $x_{dc,l}$ can be written as follows [24]:

(1) $$x_{dc,l}=\sum_{i=1}^{N_{c}}\xi_{l+l_{d},i}v\left(\omega_{d,i},\vartheta_{i}\right)=\sum_{i=1}^{N_{c}}\xi_{l+l_{d},i}v_{s}\left(\vartheta_{i}\right)⊗v_{t}\left(\omega_{d,i}\right)$$

where *N*_c_ denotes the number of independent clutter patches; $\xi_{l+l_{d},i}$ denotes the complex amplitude of the *i-*th clutter patch for which its time bin is (*l + l_d_*); $\vartheta_{i}$ and $\omega_{d,i}$ are its spatial frequency and relative (relative to the Doppler frequency of DP signal) Doppler frequency, respectively; $\omega_{d,i}=\omega_{i}-\omega_{d}$ where $\omega_{i}$ and $\omega_{d}$ are the normalized Doppler frequencies of the *i-*th clutter patch and DP signal, respectively; $v\left(\omega_{d,i},\vartheta_{i}\right)$ is the MN×1 space-time steering vector with normalized Doppler frequency $\omega_{d,i}$ and the spatial frequency $\vartheta_{i}$; and $v_{s}\left(\vartheta \right)$ and $v_{t}\left(\omega \right)$ are the temporal and spatial steering vector, respectively, which are expressed as the following.

(2) $$v_{s}\left(\vartheta \right)={\left[1,\exp\left(j2\pi \vartheta \right),\cdots ,\exp\left(j2\pi \left(N-1\right)\vartheta \right)\right]}^{Τ}$$

(3) $$v_{t}\left(\omega \right)={\left[1,\exp\left(j2\pi \omega \right),\cdots ,\exp\left(j2\pi \left(M-1\right)\omega \right)\right]}^{Τ}$$

The second part is MP clutter. Correspondingly, when scatterer echoes with time bin (*l + l_p_*) are passed through the matched filtering with the *p-*th (*p* = 1, 2, …, *N_T_*) MP signal, the *p-*th MP clutter snapshot of the *l-*th range cell can be expressed as the following:

(4) $$x_{pc,l}=\varepsilon_{p}\sum_{i=1}^{N_{c}}\xi_{l+l_{p},i}v\left(\omega_{p,i},\vartheta_{i}\right)=\varepsilon_{p}x_{dc,l+\left(l_{p}-l_{d}\right)}⊙g\left(\omega_{p}-\omega_{d}\right)$$

where $\varepsilon_{p}$ is the relative (relative to the amplitude of DP signal) complex amplitude of the *p-*th MP signal [24]; $\xi_{l+l_{p},i}$ denotes the complex amplitude of the *i-*th clutter patch for which its time bin is (*l + l_p_*); $\omega_{p,i}$ is the relative (relative to the Doppler frequency of the *p-*th MP signal) Doppler frequency; $\omega_{p,i}=\omega_{i}-\omega_{p}$ where $\omega_{i}$ and $\omega_{p}$ are the normalized Doppler frequency of the *i-*th clutter patch and the *p-*th MP signal, respectively; and the modified space-time steering vector is $g\left(\omega_{p}-\omega_{d}\right)=1_{N\times 1}⊗v_{t}\left(\omega_{p}-\omega_{d}\right)$.

Therefore, the received clutter-plus-noise snapshot for the *l-*th range cell is modeled as the following:

(5) $$x_{l}=x_{dc,l}+\sum_{p=1}^{N_{T}}x_{pc,l}+x_{n}$$

where the noise vector $x_{n}$ is assumed to be Gaussian and spatially and temporally white. Since the three components in Equation (5) are assumed to be mutually uncorrelated [24], the space-time covariance matrix is given by the following:

(6) $$R_{x}=E\left[x_{l}x_{l}^{H}\right]=R_{d}+\sum_{p=1}^{N_{T}}R_{p}+R_{n}$$

where $R_{d}=E\left[x_{dc,l}x_{dc,l}^{H}\right]$, $R_{p}=E\left[x_{pc,l}x_{pc,l}^{H}\right]$, and $R_{n}=E\left[x_{n}x_{n}^{H}\right]$ denote DP clutter, the *p-*th MP clutter, and thermal noise covariance matrix, respectively. Under the principle of linearly constrained minimum variance, the optimal STAP weight is given by the following:

(7) $$w_{x}=\mu R_{x}^{-1}v\left(\omega_{t},\vartheta_{t}\right)$$

where μ is the normalized constant; $\omega_{t}$ and $\vartheta_{t}$ are the normalized Doppler frequency and spatial frequency of the hypothetic target, respectively; $v\left(\omega_{t},\vartheta_{t}\right)=v_{s}\left(\vartheta_{t}\right)⊗v_{t}\left(\omega_{t}\right)$ is its space-time steering vector. In practice, the unknown covariance matrix $R_{x}$ is usually estimated from $L_{1}$ training samples, i.e., ${\hat{R}}_{x}=1/L_{1}\sum_{i=1,i\neq l}^{L_{1}+1}x_{i}x_{i}^{H}$.

The above analyses show that MP clutter exists in the received snapshots when the reference signal is contaminated by the MP signals. As a result, the background interference is disturbed, and the estimated covariance matrix is corrupted [24]. In order to achieve the target detection, both MP clutter and DP clutter need to be canceled. However, directly using STAP requires more degrees of freedom than suppressing DP clutter alone. Additionally, targets that fall within the MP clutter area will also be suppressed as well in STAP.

CM, which cancels MP clutter prior to the DP clutter suppression by STAP, can overcome these issues. The existing CMs use SRAs for MP clutter estimation [24,25], where the range-Doppler dictionary is exploited to estimate MP clutter and eliminate its influence on STAP performance. However, a bias between the real MP clutter and the predefined discrete range-Doppler grids is hardly avoided in practice, which results in an imprecise estimation of MP clutter and a performance degradation on target detection. Therefore, it is crucial to develop a MP clutter suppression method for off-grid effects mitigation in airborne passive radar.

## 3. The Proposed CM for MP Clutter Suppression and Off-Grid Effects Mitigation

In this section, we provide a brief introduction to the existing CMs, describe the proposed MP clutter suppression method for off-grid effects mitigation, and detail the CM based on the proposed algorithm.

### 3.1. Review of the Exiting CMs

The existing CMs can effectively suppress the MP clutter and DP clutter in the absence of off-grid effects. It is concluded from [24] that MP clutter is related to two factors, which include the received space-time snapshots and the modified space-time steering vectors. Thus, the Doppler frequency plane is uniformly discretized into *Q* grid points. Let $\omega_{q}$ and $g\left(\omega_{q}\right)=1_{N\times 1}⊗v_{t}\left(\omega_{q}\right)$ (q=1,2,…,Q) be the discretizing Doppler frequency and the corresponding modified space-time steering vector, respectively. Assuming that MP clutter can be represented by *D* snapshots, the MP clutter snapshot at range cell *l* shown in Equation (4) can be rewritten as follows [24]:

(8) $$\sum_{p=1}^{N_{T}}x_{pc,l}=S_{l}\alpha$$

where α denotes the DQ×1 profile with nonzero elements representing the MP clutter; the MN×DQ range-Doppler dictionary is $S_{l}=\left[S_{D,l}⊙G_{1},S_{D,l}⊙G_{2},\ldots ,S_{D,l}⊙G_{Q}\right]$; the MN×D range dictionary matrix is constructed by the snapshots from range cell *l* + 1 to *l* + *D*, i.e.,$S_{D,l}=\left[x_{l+1},x_{l+2},\ldots ,x_{l+D}\right]$; and the modified space-time steering matrix is $G_{q}=1_{1\times D}⊗g\left(\omega_{q}\right)=1_{N\times D}⊗v_{t}\left(\omega_{q}\right)$, q=1,2,…,Q. Since there is a degree of the sparsity in the profile α [24], the above equation is also called the sparse measurement model of MP clutter in what follows.

Under the assumption that MP clutter is perfectly located at the discretized range-Doppler grids, the MP clutter suppression problem can be transformed into the following optimization problem [24]:

(9) minαJ1(α)=minαE{‖xl−Slα‖22}+2κ‖α‖1+2Re{ςα}

where κ is a sparse constant parameter, $\varsigma =\sigma^{2}1_{1\times MN}\Phi$, $\sigma^{2}$ is the noise power per element, and the MN×DQ matrix $\Phi =\left[G_{1},G_{2},\ldots ,G_{Q}\right]$.

Let the gradient term of the above equation with respect to $\alpha^{*}$ be zero, we can obtain the following:

(10) $$\alpha =R_{S}^{-1}\left(r_{Sx}-\kappa \mathrm{sign}\left(\alpha \right)+\varsigma^{H}\right)$$

where the cross-correlation vector is $r_{Sx}=E\left[S_{l}^{H}x_{l}\right]$; the covariance matrix $R_{S}=E\left[S_{l}^{H}S_{l}\right]$; and sign(⋅) is a component-wise function.

Since α exists on both sides of Equation (10), it is not an explicit expression for α. To obtain a solution, SRAs are used to address the above optimization problem. As a result, the MP clutter is estimated effectively under the condition that MP clutter is perfectly located on the discretizing grid points. When this assumption is violated, the MP clutter suppression performance will degrade, which restricts the applicability of the existing algorithms presented in [24,25]. Therefore, it is necessary to derive a novel MP clutter suppression method to eliminate off-grid effects.

### 3.2. The Proposed CM for Clutter Suppression and Off-Grid Effects Mitigation

In this subsection, we derive a MP clutter suppression algorithm for off-grid effects mitigation. Then, the CM based on the proposed algorithm is described, and the overall procedure of the proposed approach is summarized.

Based on the above development, the question that arises now is how to eliminate the mismatch between the range-Doppler atoms and the real MP clutter. To overcome this issue, the MP clutter estimation method based on local search is proposed. In the proposed algorithm, the range-Doppler atom is selected iteratively, and a global search and several local searches are performed in each iteration. For the sake of simplicity, let symbol (t) be the *t-*th iteration, and let symbols (t,0) and (t,k)(k=1,2,…) be the global search and the *k-*th local search of the *t-*th iteration, respectively.

First, the Doppler frequency plane is uniformly discretized into *Q* grid points with a relatively large Doppler frequency interval Δω, such as the method presented in [24,25]. Thus, the global dictionary $S_{l}$ is obtained with the modified snapshot $d_{l,\gamma }$ being the γ-th (γ=1,2,…,DQ) global atom, given by the following.

(11) $$\begin{array}{ll} S_{l} & =\left[x_{l+1}⊙g\left(\omega_{1}\right),\ldots ,x_{l+D}⊙g\left(\omega_{1}\right),\ldots ,x_{l+1}⊙g\left(\omega_{Q}\right),\ldots ,x_{l+D}⊙g\left(\omega_{Q}\right)\right]\\ & =\left[d_{l,1},\ldots ,d_{l,D},\ldots ,d_{l,\left(Q-1\right)D+1},\ldots ,d_{l,DQ}\right] \end{array}$$

Then, we can obtain global responses by computing the inner product via the matrix-vector multiplication, which is similar to the orthogonal matching pursuit algorithm [16]. Thus, the global response $a_{\gamma }\left(t,0\right)$ of the γ-th (γ=1,2,…,DQ) global atom at the *t-*th iteration is given by the following:

(12) $$a_{\gamma }\left(t,0\right)=E\left[d_{l,\gamma }^{H}h_{l}\left(t\right)\right]$$

where $h_{l}\left(t\right)$ denotes the residual vector of the *l-*th range cell at the *t-*th iteration, which is initialized as $h_{l}\left(1\right)=x_{l}$; and $E\left[d_{l,\gamma }^{H}h_{l}\left(t\right)\right]$ is estimated by the *L*_2_ training samples in practice, i.e., $a_{\gamma }\left(t,0\right)=1/L_{2}\sum_{i=1}^{L_{2}}d_{i,\gamma }^{H}h_{i}\left(t\right)$. Consequently, the global index at the *t-*th iteration is picked up by the following.

(13) $$\gamma \left(t,0\right)=\arg\underset{\gamma \in \left\{1,2,\ldots ,DQ\right\}}{\max}\left[a_{\gamma }\left(t,0\right)\right]$$

The corresponding atom is defined as the optimal global atom at the *t-*th iteration, i.e., $d_{l,\gamma \left(t,0\right)}$. The range cell and normalized Doppler frequency of the optimal global atom $d_{l,\gamma \left(t,0\right)}$ are expressed as τ(t) and ω(t,0), respectively.

**Figure 2:**
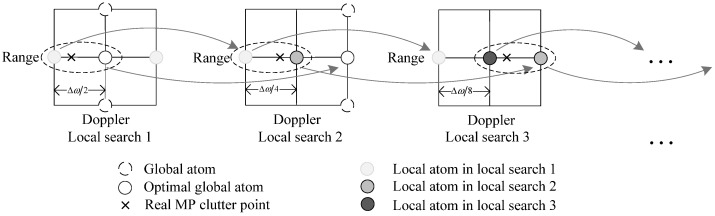
Illustration of the local dictionary construction.

After the global search, several local searches are performed. The local range-Doppler dictionary is designed with the optimal global atom, as shown in Figure 2.

In the first local search of the *t-*th iteration, we exploit the optimal global atom to construct the local dictionary. With the optimal global atom as the center, three local atoms are obtained around the Doppler frequency dimension where the Doppler frequency interval is Δω/2. Thus, we can obtain the first local dictionary of the *t-*th iteration $S_{l}\left(t,1\right)$, given by the following:

(14) $$\begin{array}{l} S_{l}\left(t,1\right)=\left[x_{l+\tau \left(t\right)}⊙g\left(\omega \left(t,0\right)-\frac{\Delta \omega }{2}\right),x_{l+\tau \left(t\right)}⊙g\left(\omega \left(t,0\right)\right),x_{l+\tau \left(t\right)}⊙g\left(\omega \left(t,0\right)+\frac{\Delta \omega }{2}\right)\right]\\ =\left[d_{l,1}\left(t,1\right),d_{l,2}\left(t,1\right),d_{l,3}\left(t,1\right)\right] \end{array}$$

where $d_{l,\eta }\left(t,k\right)$(η=1,2,3) denotes the η-th local atom in the *k-*th local search of the *t-*th iteration. Correspondingly, the local response of $d_{l,\eta }\left(t,1\right)$(η=1,2,3) is calculated by the following.

(15) $$a_{\eta }\left(t,1\right)=\frac{1}{L_{2}}\sum_{i=1}^{L_{2}}d_{i,\eta }^{H}\left(t,1\right)h_{i}\left(t\right)$$

Subsequently, the local atoms with the largest and the second largest response in Equation (15) are selected, which is used as edges in the second local search. Based on that, three atoms are obtained to construct the second local dictionary with the Doppler frequency interval Δω/4, as shown in Figure 2. Then, the local response is computed again, and the selected local atoms are updated; the above operation is repeated. Consequently, by using the two selected local atoms in the (*k*−1)-th local search as edges, the *k-*th local dictionary $S_{l}\left(t,k\right)=\left[d_{l,1}\left(t,k\right),d_{l,2}\left(t,k\right),d_{l,3}\left(t,k\right)\right]$ is constructed with the Doppler frequency interval $\Delta \omega /2^{k}$. Therefore, the ηth(η=1,2,3) local response $a_{\eta }\left(t,k\right)$ at the *k-*th local search of the *t-*th iteration is calculated by the following.

(16) $$a_{\eta }\left(t,k\right)=\frac{1}{L_{2}}\sum_{i=1}^{L_{2}}d_{i,\eta }^{H}\left(t,k\right)h_{i}\left(t\right)$$

The above operation is repeated until the gain in response of the *k-*th local search, defined as $\max\left\{a_{\eta }\left(t,k\right)-a_{\eta }\left(t,k-1\right)\right\}$, is less than the local threshold $\delta_{\eta }$. Assuming that $K_{t}$ denotes the index of the last local search of the *t-*th iteration, the optimal atom ${\hat{d}}_{l}\left(t\right)$ is obtained by selecting the index corresponding to the largest response $a_{\eta }\left(t,K_{t}\right)$ (η=1,2,3), given by the following:

(17) $$\{\begin{array}{c} \eta \left(t,K_{t}\right)=\arg\underset{\eta \in \left\{1,2,3\right\}}{\max}\left(a_{\eta }\left(t,K_{t}\right)\right)\\ {\hat{d}}_{l}\left(t\right)=d_{l,\eta \left(t,K_{t}\right)}\left(t,K_{t}\right) \end{array}$$

and the normalized Doppler frequency of the *t-*th optimal atom ${\hat{d}}_{l}\left(t\right)$ is expressed as $\hat{\omega }\left(t\right)$.

The *t-*th iteration has been completed now. Then, the MP clutter profile is updated, and the residual vector is calculated. Let ${\hat{S}}_{l}$ be the optimal dictionary assembled by the selected columns of $S_{l}\left(t,K_{t}\right)$, and ${\hat{S}}_{l}$ is initialized as ${\hat{S}}_{l}\left(0\right)=ø$. Thus, ${\hat{S}}_{l}$ at the *t-*th iteration is updated by the following:

(18) $${\hat{S}}_{l}\left(t\right)=\left[{\hat{S}}_{l}\left(t-1\right),{\hat{d}}_{l}\left(t\right)\right]$$

and the MP clutter profile at the *t-*th iteration is given by the following:

(19) $$\hat{\alpha }\left(t\right)=R_{\hat{S}}^{-1}\left(t\right)\left[r_{\hat{S}x}\left(t\right)+{\hat{\varsigma }}^{H}\left(t\right)\right]$$

where $\hat{\varsigma }\left(t\right)=\sigma^{2}1_{1\times MN}\hat{\Phi }\left(t\right)$,$\hat{\Phi }\left(t\right)=\left[\hat{\Phi }\left(t-1\right),g\left(\hat{\omega }\left(t\right)\right)\right]$ in which $\hat{\Phi }$ is initialized as $\hat{\Phi }\left(0\right)=Ø$; the covariance matrix $R_{\hat{S}}\left(t\right)$ and the cross-correlation vector $r_{\hat{S}x}\left(t\right)$ at the *t-*th iteration are as follows.

(20) $$R_{\hat{S}}\left(t\right)=\frac{1}{L_{2}}\sum_{i=1}^{L_{2}}{\hat{S}}_{i}^{H}\left(t\right){\hat{S}}_{i}\left(t\right)$$

(21) $$r_{\hat{S}x}\left(t\right)=\frac{1}{L_{2}}\sum_{i=1}^{L_{2}}{\hat{S}}_{i}^{H}\left(t\right)x_{i}$$

Moreover, the residual vector of the *l-*th range cell is updated by the following.

(22) $$h_{l}\left(t+1\right)=x_{l}-{\hat{S}}_{l}\left(t\right)\hat{\alpha }\left(t\right)$$

The iteration terminates when a certain criterion is satisfied. For example, the gain in signal-to-interference-plus-noise-ratio (SINR) loss of the *t-*th iteration, which is defined as I(t)−I(t−1) where I(t) denotes the SINR loss of the *t-*th iteration, is smaller than a predetermined iteration threshold δ. In this paper, the gain performances are used as the stopping criterion in local search and iteration. The reason is that the convergence can be determined by using a simple method: if $a_{\eta }\left(t,k\right)\approx a_{\eta }\left(t,k-1\right)$ or I(t)≈I(t−1), the algorithm will converge. This method is also adopted in [32,33]. Here, the gain performance in SINR loss or response denotes the change of I(t) or $a_{\eta }\left(t,k\right)$ from I(t−1) or $a_{\eta }\left(t,k-1\right)$. Then, the user should impose a threshold in the programming, which can be determined by considering the computational complexity and the MP estimation performance. Consequently, the local search or iteration will terminate if the gain value is smaller than the threshold.

Based on the above development, the estimated MP clutter profile $\hat{\alpha }$ and sub-dictionary ${\hat{S}}_{l}$ are obtained.

Now, the proposed cascaded suppression method with off-grid effects mitigation in airborne passive radar can be described as the following two-step suppression process.

First, the proposed algorithm is used for MP clutter suppression. By using the derived ${\hat{S}}_{l}$ and $\hat{\alpha }$, the output snapshot at the *l-*th range cell is calculated by the following.

(23) $$y_{l}=x_{l}-{\hat{S}}_{l}\hat{\alpha }$$

Then, DP clutter is canceled by the existing STAP algorithm. The scalar output of the proposed CM is given by the following:

(24) $$z_{l}=w_{y}^{H}y_{l}=w_{y}^{H}\left(x_{l}-{\hat{S}}_{l}\hat{\alpha }\right)$$

where $w_{y}$ is the STAP weight vector.

The overall procedure of CM based on the proposed algorithm is presented in Algorithm 1.
**Algorithm 1:** The proposed CM for off-grid effects mitigation
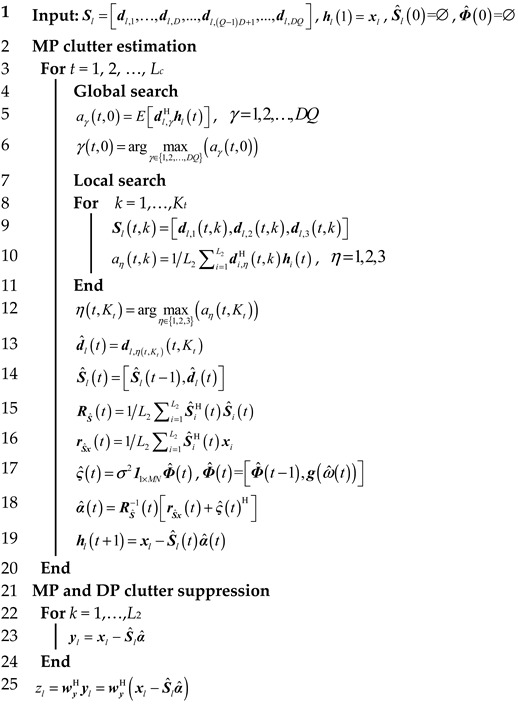


### 3.3. Analyses of Computational Complexity

In this subsection, we discuss the computational complexity of the proposed method in terms of the number of additions and multiplications. In the *t-*th iteration, $L_{2}MNDQ$ multiplications and $L_{2}DQ\left(MN-1\right)$ additions are required to compute γ(t,0) in the global search; $3MNK_{t}\left(L_{2}+1\right)$ multiplications and $3L_{2}K_{t}\left(MN-1\right)$ additions are required to compute $\eta \left(t,K_{t}\right)$ in local search; $ο\left(t^{3}\right)+t^{2}\left(MNL_{2}+1\right)+MN\left(tL_{2}+t+1\right)$ multiplications and $ο\left(t^{3}\right)+t^{2}\left(MNL_{2}-L_{2}+1\right)+t\left(MN-1\right)\left(L_{2}+1\right)$ additions are required to compute $\hat{\alpha }\left(t\right)$; and tMN multiplications and tMN additions are required to compute $h_{l}\left(t+1\right)$. Therefore, in the *t-*th iteration of proposed CM, $ο\left(t^{3}\right)+t^{2}+MNL_{2}\left(3K_{t}+DQ+t^{2}+t\right)+MN\left(3K_{t}+2t+1\right)$ multiplications and $ο\left(t^{3}\right)+L_{2}\left(MN-1\right)\left(DQ+3K_{t}+t^{2}+t\right)+t\left(t+2MN-1\right)$ additions are required. Although the added local search step slightly increases computational complexity, the proposed algorithm selects more accurate atoms than compared to merely applying the global searches in the presence of off-grids effects, which will be verified in the next section.

## 4. Simulations and Performance Analyses

In this section, simulation experiments are provided to validate the theorical derivation and demonstrate the performance of the proposed method. In the simulation, a digital video broadcasting transmitter is utilized as the illustrator of opportunity in the airborne passive system. In this paper, a Gaussian random signal is used as an approximate model for the transmitted signal [24]. Some important simulation parameters are listed in Table 1. In the simulation, the noise power is normalized, and the direct-path signal to noise ratio (DNR) is set to 60dB [34]. In the reference signal, the normalized Doppler frequency of DP signal is 0.5, which corresponds to Doppler frequency 200 Hz. There are three MP signals in the reference signal. Their relative (relative to the time bin of the DP signal) time bins are three, four, and nine, respectively. The corresponding normalized Doppler frequencies are −0.25, −0.18, and −0.33, respectively. The corresponding MP signal to DP signal ratios (MDRs) are set to −18 dB, −18 dB, and −25 dB, respectively. We set Q=11, and zero vectors are used to initial the MP clutter suppression weight vector. All presented results are averaged over 100 independent Monte Carlo runs.

### 4.1. Setting of the Number of Snapshots in Range Dictionary

**Figure 3:**
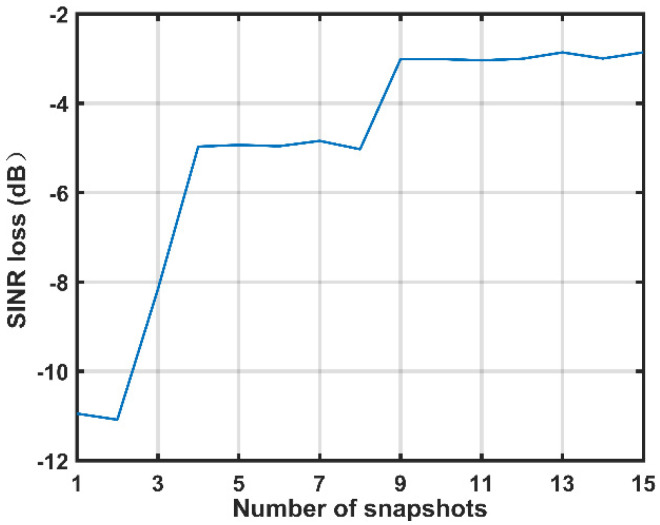
SINR loss performance of the proposed method against the number of snapshots.

In the first experiment, we examine the SINR loss performance of the proposed method against the number of snapshots in range dictionary (the value of *D*). Simulation results are shown in Figure 3. It can be observed that the SINR loss performance would degrade when the value of *D* is too small. This is because the atoms in the proposed global dictionary cannot match the real MP clutter accurately, which results in the error of the estimation of MP clutter. On the other hand, when *D* is set to a large value (D>9), the SINR loss performance will not improve much. Additionally, the larger the value of *D*, the larger the global dictionary dimension will be and, in turn, the higher the computational complexity. Therefore, the number of snapshots should be as few as possible but should still ensure estimation performance, which means *D* is selected to obtain a trade-off between the SINR loss performance and the computational complexity. Based on the simulation result, we set D=9 in the following simulations.

### 4.2. Setting of the Iteration Threshold

**Figure 4:**
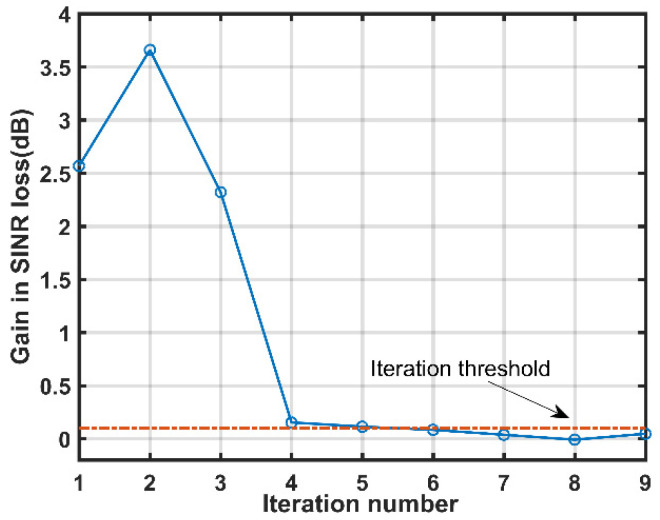
Gain performance in SINR loss versus iteration number.

Figure 4 provides the gain performance in SINR loss of the proposed method against the iteration number. It is observed from Figure 4 that the gain in SINR loss becomes closer to zero with the increment of iteration number. This phenomenon can be explained as follows: when the iteration number is set to a small value, more important atoms are selected to estimate MP clutter with the increased iteration number, which results in the improvement in SINR loss performance; however, if the iteration number exceeds six, atoms that correspond to insignificant MP clutter component would be incorporated into the estimated dictionary. In this case, the SINR loss performance is not improved much but at the cost of a higher computational cost. Therefore, the iteration threshold δ can provide a trade-off between SINR loss performance and computational complexity. Based on the above analyses and simulation results, we set the iteration threshold for the iteration stopping criterion as 0.1 in the following simulations, which is denoted by the horizontal dotted line in Figure 4. Consequently, the iteration terminates when the iteration number equals six.

### 4.3. Setting of the Local Threshold

**Figure 5:**
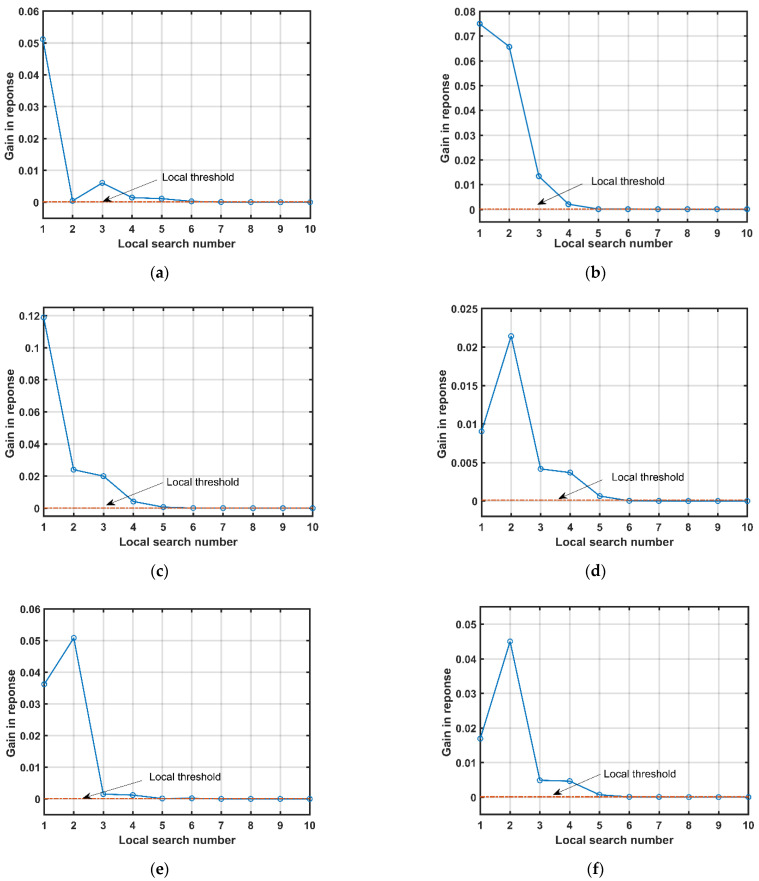
Gain performance in response against the number of local searches. (**a**) In the first iteration; (**b**) in the second iteration; (**c**) in the third iteration; (**d**) in the fourth iteration; (**e**) in the fifth iteration; (**f**) in the sixth iteration.

The third experiment is set up to examine the local search performance of each iteration. In each iteration, the local search terminates when the gain in response is less than the local threshold. Figure 5 provides the gain performances in response against the number of local searches. As can be observed from Figure 5, as the number of local searches increases, the gain in response is closer to zero in each iteration, which means the selected atom is closer to the MP clutter component. However, when the number of local searches is set to a large value, the response performance will not improve much, and in turn the computational complexity is high. Therefore, setting a suitable local threshold is important in order to obtain a trade-off between the response performance and computational cost. Based on the simulation results, the local threshold $\delta_{\eta }$ is set to 0.0001 in the following simulations (the horizontal dotted line in Figure 5 indicates the local threshold). Consequently, the numbers of local search in six iterations are seven, seven, six, six, seven, and six, respectively.

### 4.4. Distribution of Selected Atoms in the Range-Doppler Plane

In the fourth experiment, the distribution of selected atoms obtained via the proposed algorithm is compared with the real atoms related to MP clutter component. Here, MP clutter component consists of the true MP clutter and their derived ghost MP clutter defined as sub-MP clutter in [24]. Since the MDRs of true MP signals are −18 dB, −18 dB, and −25 dB, respectively, and the corresponding time bins-normalized Doppler frequencies are [3, 0.25], [4, 0.18], and [9, 0.33], respectively, the time bins-normalized Doppler frequencies of three significant ghost MP clutter are [6, 0.5], [7, 0.43] and [8, 0.36], respectively. The range cells and normalized Doppler frequencies of the real atoms in six iterations are listed in Table 2.

**Figure 6:**
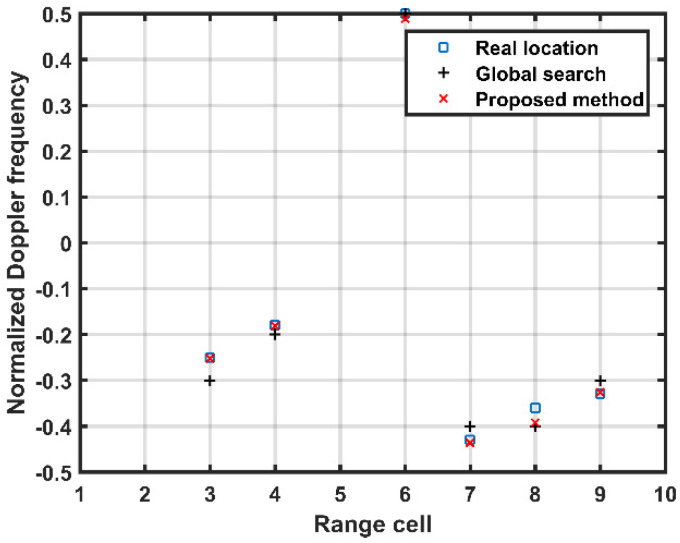
Distribution of selected atoms in the range-Doppler plane.

Figure 6 provides the distribution of the selected atoms in the range-Doppler plane of the proposed method. The square denotes the real location of the atoms. From Figure 6, it is observed that the atoms selected by the global searches deviate from the real location severely, while those obtained from the proposed method are close to the real location. This verifies that the additional local search steps of the proposed method can obtain a more accurate estimate performance of the MP clutter than merely applying the global searches.

### 4.5. Comparison with the Existing CM

In the fifth experiment, the performances of the proposed method are compared with those obtained from the existing method. As existing CMs based on SRAs exploit the same measurement model and optimization problem, the performance degradation caused by the off-grid problem is almost identical in all existing CMs. Additionally, compared with other two exiting CMs, the CM using *L*_1_-EFWLMS can achieve a trade-off between the computational complexity and SINR loss performance. Therefore, for a fair and clear comparison, the existing CM using *L*_1_-EFWLMS is selected for comparison, and its performances are discussed in what follows. For the CM using *L*_1_-EFWLMS, the Doppler frequency plane is discretized into 11 grids, the length of the sliding-window is 4, and the value of the parameter *D* is set to 9. It is observed that there is a bias between the real MP clutter and the predefined discrete range-Doppler grids, which means that the off-grid issues exist in the CM using *L*_1_-EFWLMS. In the following simulations, we denote the CM using *L*_1_-EFWLMS as the existing CM for simplicity. The number of training snapshots used to estimate the clutter covariance matrix is 200 for all aforementioned methods.

**Figure 7:**
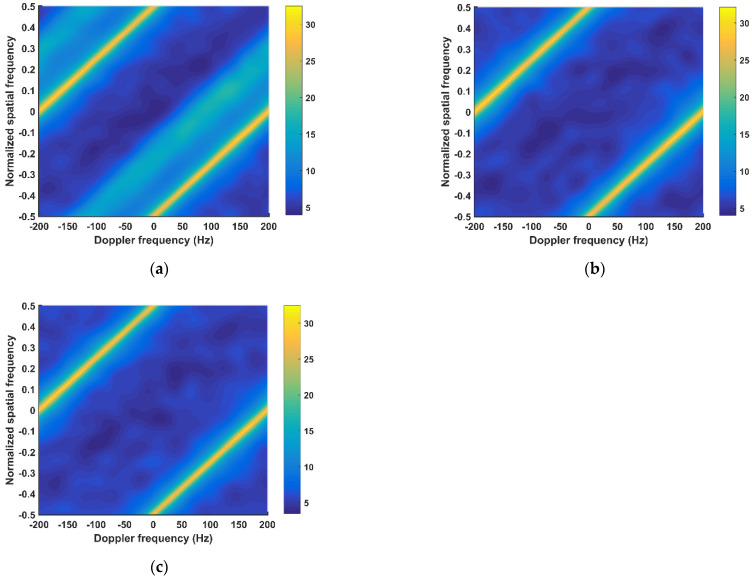
Clutter power spectrum. (**a**) STAP; (**b**) the existing CM; (**c**) the proposed CM.

Figure 7 shows the clutter power spectra corresponding to STAP, the existing CM, and the proposed CM. Since no MP clutter suppression algorithm is utilized in STAP, the power spectrum exhibits the distributions of DP clutter and MP clutter for STAP, as indicated by the Figure 7a. Conversely, the proposed CM and the existing CM have an extremely low power in MP clutter area; however, they maintain a similar power as STAP in the DP clutter area. Thus, the clutter power spectra of these two CMs exhibit slight expansions. It verifies that the proposed algorithm can suppress the MP clutter and eliminate its influence on STAP effectively.

**Figure 8:**
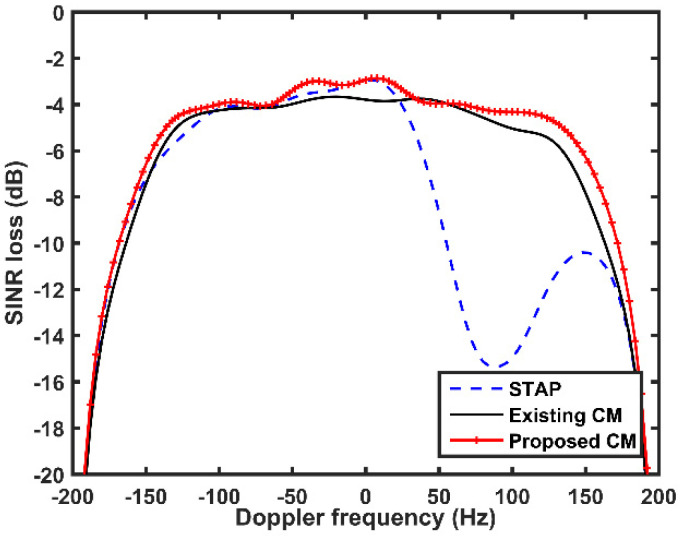
SINR loss performances of different methods.

Then, we illuminate the SINR loss performances of different methods along the main beam direction. The simulation results are presented in Figure 8. The Doppler frequencies span from −200 to 200 Hz. As can be observed from Figure 8, STAP has nulls in both MP clutter area (corresponds to the Doppler frequencies of 72 Hz, 100 Hz, and 132 Hz) and DP clutter area (corresponds to the Doppler frequencies of 200 Hz and −200 Hz), which indicates that the detection performance of STAP deteriorates for a target located in the MP clutter area. Additionally, for the existing CM and the proposed CM, nulls in DP clutter area are similar to STAP while satisfactory SINR loss performance is obtained in the MP clutter area. This indicates that these two CMs can achieve MP clutter suppression before STAP and improve detection ability. Particularly, the proposed CM exhibits an enhanced SINR loss performance than the existing CM. The superiority of the proposed CM lies in the fact that it can eliminate the influence of the off-grid issues and provide more accurate MP clutter estimation than the exiting CM due to the additional local search steps. This verifies that the proposed off-grid algorithm can obtain better MP clutter suppression performance than the exiting CM in the presence of off-grid problem.

**Figure 9:**
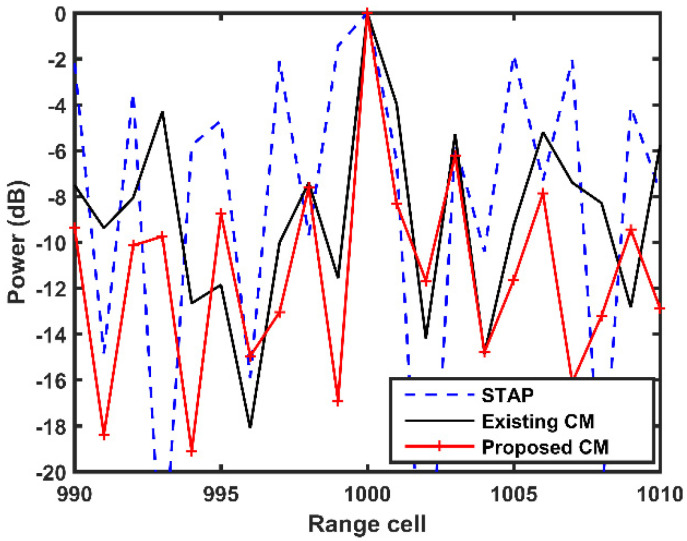
Target detection results of different methods.

Next, we focus on the target detection performance of the proposed CM. The simulation results are depicted in Figure 9. There is a target in the 1000th range cell with a relative (relative to the Doppler frequency of the DP signal) Doppler frequency of 100Hz. Snapshots from 990th to 1010th range cells are filtered by STAP, the existing CM, and the proposed CM. As it can be observed, STAP no longer performs properly since the target is located the MP clutter area, which is coincident with the conclusions presented in Figure 8. Additionally, the proposed CM can provide more desirable detection performance than the exiting CM. Thus, the simulation results verify the effectiveness of the proposed CM in the presence of the off-grid problem.

**Figure 10:**
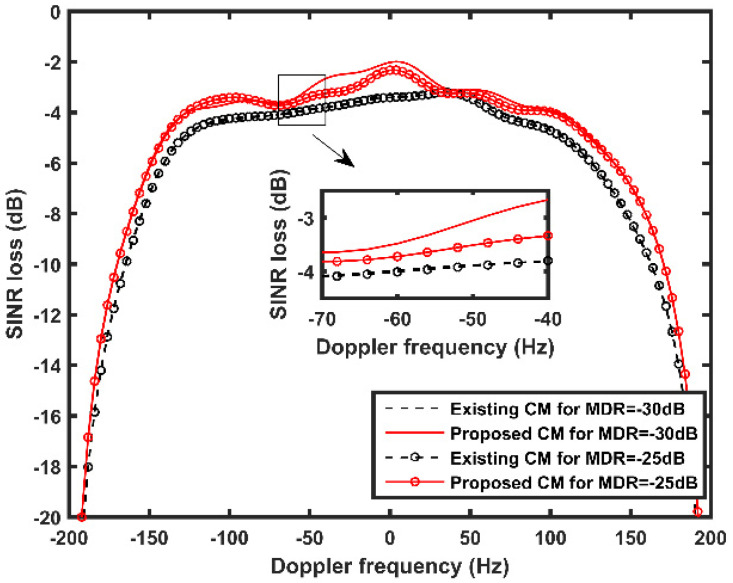
SINR loss performances with different MDR values.

In the sixth experiment, the SINR loss performances and target detection results of the proposed CM are compared with that of the existing CM in different MDR scenarios. Figure 10 depicts the simulation results considering two MDR cases, which is MDR = −25 dB and MDR = −30 dB. Other simulation parameters are the same as the fifth experiment. It is observed that the proposed CM features a better SINR loss performance than the existing CM in both cases. This verifies that, considering the off-grids effects, the proposed CM can suppress MP clutter effectively, even in different MDRs.

**Figure 11:**
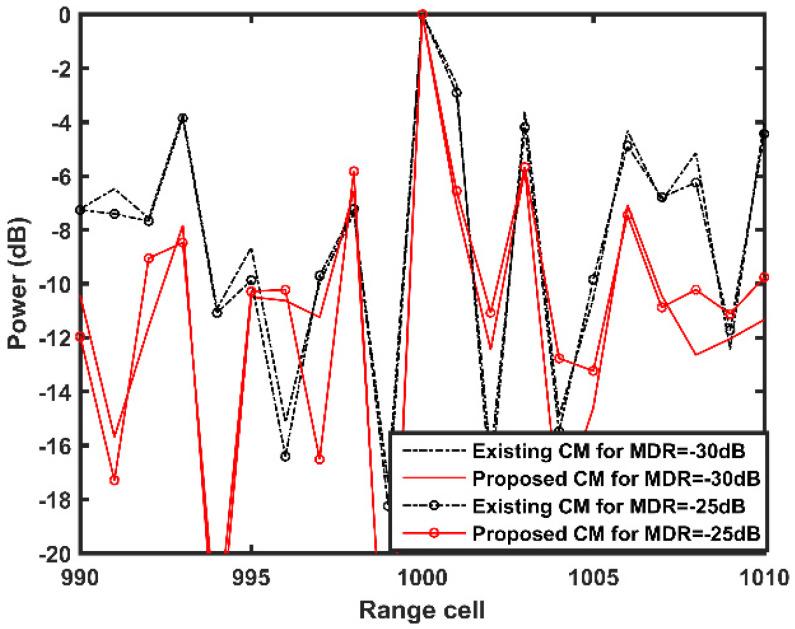
Target detection results with different MDR values.

In Figure 11, the corresponding target detection results are presented. Similar to Figure 10, the proposed CM features a better performance in target detection than the existing CM in both cases. This verifies that a bias between the MP clutter and the discretizing grid points results in performance degradation in the existing CM; however, the local search steps of the proposed CM can effectively eliminate its effect in these two cases.

In the seventh experiment, we make a quantitative comparison between two CMs with different MDR values. The SINR loss value at the target Doppler frequency is summarized in Table 3. Three MP signals with the same MDR are considered. MDR value is set to −15 dB, −20 dB, −25 dB, −30 dB, −35 dB, and −40 dB, respectively. The Doppler frequencies of the MP signals are assumed to follow a uniform distribution. Notably, the Doppler frequencies of the MP signals follow a uniform distribution within [−0.5, 0.5]. Other simulation parameters are the same as the fifth experiment. It is clear that, regardless of the MDR value, the proposed CM outperforms the existing CM in the presence of off-grid issues.

**Figure 12:**
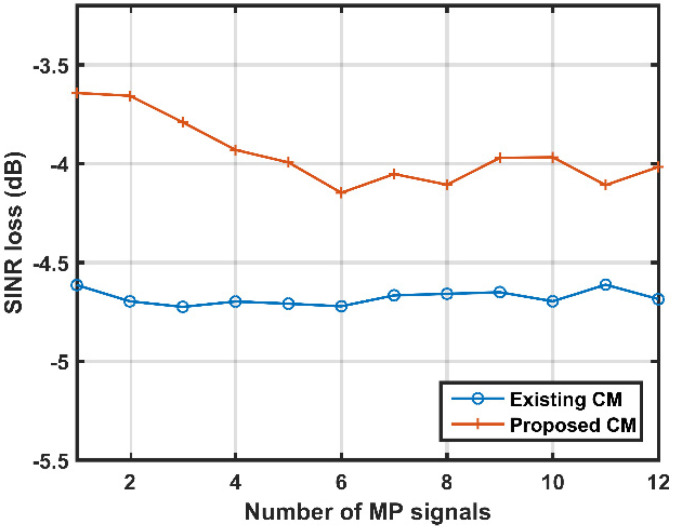
SINR loss performances of different methods against the number of MP signals.

The last experiment is set up to examine the SINR loss performances of different methods against the number of MP signals. The number of MP signals is set to 1, 2, 3,..., and 12, respectively. The Doppler frequencies and MDRs of the MP signals are both assumed to follow a uniform distribution. Notably, the Doppler frequencies and MDRs of the MP signals follow a uniform distribution within [−0.5, 0.5] and [–40, 25], respectively. Simulation results are presented in Figure 12. It is observed that the proposed CM always exhibits a better SINR loss performance than the existing CM when the number of MP signals varies. It is concluded from the above-mentioned simulation results that the proposed CM can address the off-grid problem effectively and obtain the desirable performances, regardless of the number of MP signals and the MDR values.

## 5. Conclusions

In this study, a novel MP clutter suppression method with off-grid effects mitigation is proposed in airborne passive radars with contaminated reference signals. The proposed algorithm exploits the sparse measurement model in order to construct the global dictionary and select the global atoms, to design the local dictionary in order to match the real MP clutter points, and to suppress MP clutter from all matched atoms. Due to the additional local search steps, the proposed algorithm achieves better suppression performance than compared to the existing SRA in the presence of the off-grid problem. The performances of the proposed algorithm are tested and compared with those of SRA. The results show that, considering the off-grid effects, CM based on the proposed algorithm outperforms CM based on SRA in the SINR loss performance, regardless of the number of MP signals and the MDR values.

In the future, we will extend our model to incorporate more realistic physical effects, such as channel mismatch, coherent jammers or noise-like jammers [35,36,37], and useful signal and interference mismatch [38].

## Figures and Tables

**Table 1:** Important simulation parameters.

Parameter	Value
Number of elements	10
Number of equivalent pulses in a CIT	10
Main beam look direction	Side-looking
Equivalent PRF	400 Hz
Channel spacing	λ/2
Platform velocity	100 m/s
Target range cell index	1000
Target Doppler frequency	−100 Hz

**Table 2:** Parameters of the real atoms in 6 iterations.

True and Ghost MP Clutter	Ture 1	Ture 2	True 3	Ghost 1	Ghost 2	Ghost 3
Time bin	3	4	9	6	7	8
Normalized Doppler frequency	−0.25	−0.18	−0.33	0.5	−0.43	−0.36

**Table 3:** The SINR loss value at the target Doppler frequency with different MDR values.

MDR Value (dB)	−15	−20	−25	−30	−35	−40
Existing CM (dB)	−4.961	−4.721	−4.725	−4.624	−4.542	−4.519
Proposed CM (dB)	−3.946	−4.077	−3.801	−3.925	−3.981	−3.930

## Data Availability

Data sharing is not applicable to this article.
